# Sampling and pyrosequencing methods for characterizing bacterial communities in the human gut using 16S sequence tags

**DOI:** 10.1186/1471-2180-10-206

**Published:** 2010-07-30

**Authors:** Gary D Wu, James D Lewis, Christian Hoffmann, Ying-Yu Chen, Rob Knight, Kyle Bittinger, Jennifer Hwang, Jun Chen, Ronald Berkowsky, Lisa Nessel, Hongzhe Li, Frederic D Bushman

**Affiliations:** 1 Department of Microbiology, University of Pennsylvania School of Medicine, Philadelphia, PA 19104-6076 USA; 2 Division of Gastroenterology, University of Pennsylvania School of Medicine, Philadelphia, PA 19104-6076 USA; 3 Center for Clinical Epidemiology and Biostatistics, University of Pennsylvania School of Medicine, Philadelphia, PA 19104-6076 USA; 4 Department of Biostatistics and Epidemiology, University of Pennsylvania School of Medicine, Philadelphia, PA 19104-6076 USA; 5 Department of Chemistry and Biochemistry, University of Colorado at Boulder, Boulder, CO 80309-0215 USA; 6 Instituto de Ciências Biológicas, Universidade Federal de Goiás, Goiania, GO, 74001-970, Brazil; 7 Howard Hughes Medical Institute, University of Colorado at Boulder, Boulder, CO 80309-0215 USA

## Abstract

Intense interest centers on the role of the human gut microbiome in health and disease, but optimal methods for analysis are still under development. Here we present a study of methods for surveying bacterial communities in human feces using 454/Roche pyrosequencing of 16S rRNA gene tags. We analyzed fecal samples from 10 individuals and compared methods for storage, DNA purification and sequence acquisition. To assess reproducibility, we compared samples one cm apart on a single stool specimen for each individual. To analyze storage methods, we compared 1) immediate freezing at -80°C, 2) storage on ice for 24 or 3) 48 hours. For DNA purification methods, we tested three commercial kits and bead beating in hot phenol. Variations due to the different methodologies were compared to variation among individuals using two approaches--one based on presence-absence information for bacterial taxa (unweighted UniFrac) and the other taking into account their relative abundance (weighted UniFrac). In the unweighted analysis relatively little variation was associated with the different analytical procedures, and variation between individuals predominated. In the weighted analysis considerable variation was associated with the purification methods. Particularly notable was improved recovery of *Firmicutes *sequences using the hot phenol method. We also carried out surveys of the effects of different 454 sequencing methods (FLX versus Titanium) and amplification of different 16S rRNA variable gene segments. Based on our findings we present recommendations for protocols to collect, process and sequence bacterial 16S rDNA from fecal samples--some major points are 1) if feasible, bead-beating in hot phenol or use of the PSP kit improves recovery; 2) storage methods can be adjusted based on experimental convenience; 3) unweighted (presence-absence) comparisons are less affected by lysis method.

## Background

The human microbiota is composed of a vast diversity of bacterial, archaeal, and eukaryotic microorganisms, the cells of which outnumber human cells by at least a factor of 10 [1]. The human microbiota contributes metabolic diversity that aids in the digestion of foods and the metabolism of drugs, promotes development of the immune system, and competes for niches with potentially pathogenic microorganisms. Numerous diseases are associated with alterations in the gut mirobiome, including opportunistic infections such as *C. difficile *colitis and inflammatory conditions such as Crohn's disease. Many more diseases are suspected to be attributable to alterations in the gut microbiome, but definitive data are just beginning to accumulate [2-6].

Previous work has demonstrated that many factors can influence the composition of the gut microbiota, including diet, antibiotic use, disease states, and human genotype [6-13]. Further complicating such studies are uncertainties regarding how different sampling and analytical methods influence the inferred microbiome composition [8,14]. We investigate this last point here.

New deep sequencing methods provide a convenient platform for characterizing the composition of the human microbiota [4,7,8,13,15-19]. DNA samples are prepared from microbial specimens, and then analyzed using massively parallel sequencing methods such as 454/Roche pyrosequencing [20].

Here we use pyrosequencing of the bacterial 16S rRNA gene to quantify bacterial taxa [21]. The 16S rRNA gene is comprised of highly conserved regions interspersed with more variable regions, allowing PCR primers to be designed that are complementary to universally conserved regions flanking variable regions. Amplification, sequencing, and comparison to databases allow the identification of bacterial lineages and their proportions in a community [22,23]. Uncultured bacterial communities have been studied extensively using Sanger sequencing to determine 16S rRNA gene sequences, and multiple studies have helped optimize methods [24,25]. The new deep sequencing methods allow data to be acquired much more efficiently and inexpensively, but optimal methods are less well developed (for some recent work in this area see [8,14,26]).

For analysis of the human gut microbiota, both fecal samples and mucosal biopsies can be used to quantify the bacterial taxa present. Feces have been shown to contain a representative collection of the bacterial taxa from the lower gastrointestinal tract, allowing convenient sampling for characterization of the gut microbiota [5,6,27]. Linking the human microbiome to gastrointestinal disease often requires large sample sizes, so there is a need for practical specimen acquisition methods that allow analysis of large numbers of human subjects, focusing attention on methods for collecting and analyzing fecal samples. For that reason, we investigated reproducibility within a specimen, effects of storage time and temperature, and effects of lysis and DNA purification methods on the bacterial communities detected. Trends of interest often involve comparisons between individuals, so the variation due to the above factors within a specimen from a single individual was compared to the variation between subjects. We have also compared methods for 16S rDNA gene amplification and deep sequencing. With issues of sampling and analysis clarified, we are able to reinforce the finding that human subjects show drastic differences in the compositions of their gut microbiomes.

## Results

### Sample acquisition and storage

To compare methods for fecal storage and DNA preparation, ten participants were enrolled and studied, of whom 40% were female and 30% were African American (Table 1). Each participant provided a single stool specimen that was sampled multiple times and then used for DNA extraction. Samples were processed immediately (Table 2, condition 8) or were first frozen at -80°C (Table 2, conditions 1-3, 7 and 9), placed on ice for 24 hours and then frozen at -80°C (Table 2, condition 4), placed on ice for 48 hours and then frozen at -80°C (Table 2, condition 5), or placed in PSP^® ^(Invitek) buffer at room temperature for 48 hours and then frozen at -80°C (Table 2, condition 6).

**Table 1:** Characteristics of participants

Total number of participants	10
Female sex	4

Race	

Black/African-American	3

White	7

Median age (range)	26.5 years (20 - 61)

Median body mass index (range)	25.5 (19.2 - 37.4)

Current smoker	1

Stool frequency 1-2 times/day	10

Bristol stool category	

1	0

2	4

3	1

4	4

5	0

6	1

7	0

**Table 2:** Sampling methods compared in this study.

			days at -80C	
Method Identifier	Storage Method	DNA Purification Method	min	max
1	Immediately frozen (-80°C)	Qiagen Stool	2	14
2	Immediately frozen (-80°C, sampled 1 cm from sample 1)	Qiagen Stool	6	63
3	Immediately frozen (-80°C)	MoBio PowerSoil	58	72
4	4C for 24 h, then frozen (-80°C)	Qiagen Stool	1	21
5	4C for 48 h, then frozen (-80°C)	Qiagen Stool	0	12
6	PSP for 48 h, then frozen (-80°C)	PSP	0	12
7	Immediately frozen (-80°C)	Qiagen Stool (70°C)	7	7
8	Fresh	Qiagen Stool	0	0
9	Immediately frozen (-80°C)	Hot phenol with bead beating	118	137

### Cell lysis and DNA purification

Four methods were used for DNA isolation from stool. Three commercial kits were used to isolate DNA from fecal samples-- QIAamp DNA Stool Minikit, PSP Spin Stool DNA Plus Kit, and the MoBio Powersoil DNA Isolation Kit. DNA was also purified by a fourth particularly harsh method in which cells were lysed by bead beating in the presence of hot phenol and then processed with the QIAamp DNA Stool Minikit. All samples for a single individual were from a single piece of stool.

When compared to the QIAamp kit, the DNA yields from the MoBio PowerSoil kit were approximately 10-fold less whereas the yields were the greatest for the PSP kit. The yield after bead beating in hot phenol was comparable to that obtained from the standard QIAamp DNA Stool Minikit isolation. With the QIAamp kit, yields were not affected by different storage methods.

### 454/Roche pyrosequence analysis

To compare how 16S rRNA gene sequence recovery was affected by storage and purification methods, total DNA from stool samples was PCR amplified using primers targeting regions flanking the variable regions 1 through 2 of the bacterial 16S rRNA gene (V1-2), gel purified, and analyzed using the 454/Roche GS FLX technology. The V1-2 region was chosen based on published simulations [25]. Each primer set used for PCR amplification also contained an eight base DNA bar code that indexed each subject, storage method, and DNA purification method [28-30]. PCR products were pooled, and a total of 473,169 sequence reads of average length 260 bases with correct bar codes and primer sequences were obtained for 57 samples (Additional File 1).

Subsequent analysis was carried out using the QIIME pipeline [31,32]. The pipeline takes in bar coded sequence reads, separates them into individual communities by bar code, and utilizes a suite of external programs to make taxonomic assignments (e. g. RDP [23]) and estimate phylogenetic diversity. These data are used to generate taxonomic summaries and as input to UniFrac cluster analysis (described below) [33,34].

### Bacterial taxa detected

**Figure 1:**
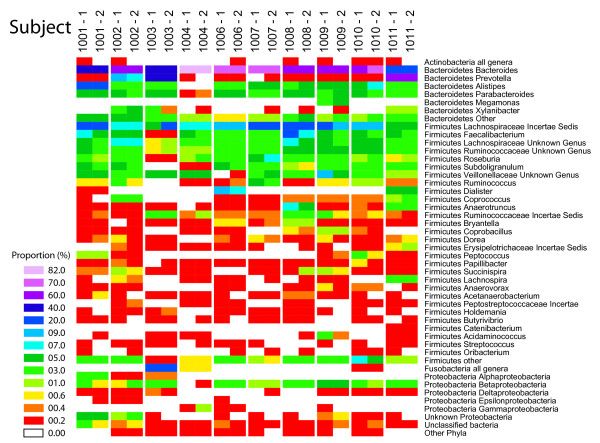
**Composition of the gut microbiome in the ten subjects studied**. Bacterial taxonomic assignments are indicated to the right of the heat map at the Phylum and Genus level except in cases where small numbers were detected (e. g. Proteobacteria), in which case taxa are summarized at higher levels. The relative abundance of each bacterial group is color coded as indicated by the key on the left (the number beside each colored tile indicates the lower bound for the indicated interval). Two samples were compared for each stool specimen, sampled on cm a part but otherwise worked up identically (conditions 1 and 2 in Table 2). The numbers of reads for the two samples from each subject were compared for significant differences using Fisher's exact test. The * indicates P < 0.05. Note that because each sequence read is treated as an individual measurement, the sample size is very large, with the result that many taxa with only modest differences nevertheless achieve significance.

Figure 1 shows the bacterial taxa detected summarized as a heat map. The most abundant genera are shown together with their Phylum-level assignments. For each subject, two identically processed samples taken 1 cm apart are shown (methods 1 and 2 in Table 2). Overall there is good reproducibility between the two adjacent "gold standard" samples--of the taxa present as greater than 1% of the total, all were detected in the paired sample. However, low abundance taxa were detected sporadically--of the samples present at 0.2%-0.4% of the total in one replicate (red in Figure 1), 35% were not detected in the second replicate. Statistical tests for significant differences are described below.

Communities were dominated by members of the *Bacteriodetes *and *Firmicute *phyla, with lower amounts of *Proteobacteria*, *Fusobacteria*, and others, as has been reported previously [5,6,27]. Pronounced differences among the subjects were evident--for example, *Fusobacteria *were particularly abundant in Subject 1003.

### Bacterial taxa recovered using the different storage and DNA isolation procedures

**Figure 2:**
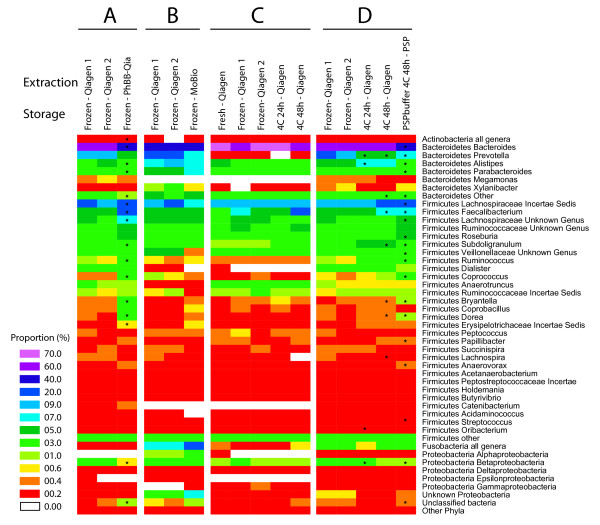
**Comparison of the recovery of different bacterial taxa with use of different stool storage and DNA isolation methods**. 473,169 sequence reads were used to characterize the 57 communities analyzed. All subjects tested for each method were pooled for comparison (summarized in Additional File 1). Methods are numbered at the top of the heat map. For the heat map scale, the number beside each colored tile indicates the lower bound for the indicated interval. Taxa are mostly indicated at the genus level; raee taxa are pooled. A) Comparison of DNA isolation using the Qiagen stool kit (methods 1 and 2) to lysis by bead-beating in hot phenol (method 9). Six subjects were compared. B) Comparison of the Qiagen stool kit samples (methods 1 and 2) to the MoBio Powersoil kit (method 3). Three subjects were compared. C) Comparison of methods for storage of stool specimens. DNA was prepared from fresh samples (method 8), samples stored frozen at -80 for several days (methods 1 and 2), or samples stored at 4°C for 24 hr (method 4) or 48 hr (method 5). Three subjects were compared. D) Comparison of stool storage in PSP (method 6) to storage methods 1, 2, 4 and 5. All 10 subjects were compared. For A) and D), the methods were compared using the Wilcoxon signed rank test to identify bacterial groups that significantly changed in proportion. (* indicates P < 0.05). Numbers of samples were too low in B) and C) for statistical testing.

The bacterial taxa recovered using the different methods are summarized in Figure 2. For each panel, all samples were pooled for subjects analyzed using each of the methods. Replicate samples (Table 2, methods 1 and 2) are included in each panel to show variation within biological replicates. Figure 2A shows that bead-beating in phenol (Table 2, method 9) led to improved recovery of some *Firmicutes *compared to the Qiagen method. Figure 2B shows that results were more similar between the MoBio method and the Qiagen method, though some differences were detected. Figure 2C shows that most of the storage methods yielded indistinguishable results, at least for proportional recovery within the major groups. Storage in PSP (Figure 2D) was associated increased proportions of several *Firmicutes*, though the increase was not as pronounced as with the phenol and beat-beating method. For both the phenol/bead-beating and PSP methods, the *Bacteriodetes *declined in abundance, likely because of the proportional increase in *Firmicutes*. Thus storage method had little effect, but use of phenol bead-beating or PSP led to increased recovery of some *Firmicutes*.

### UniFrac cluster analysis

We next sought to investigate the significance of the differences observed. In many studies of human subjects the question of interest centers on whether a biological factor (disease state, treatment, host genotype etc.) results in a measurable difference on a gut bacterial community against the background of the naturally occurring differences among humans. We thus asked whether the effects of the sample storage and DNA isolation methods were discernable against the background of variation among subjects.

**Figure 3:**
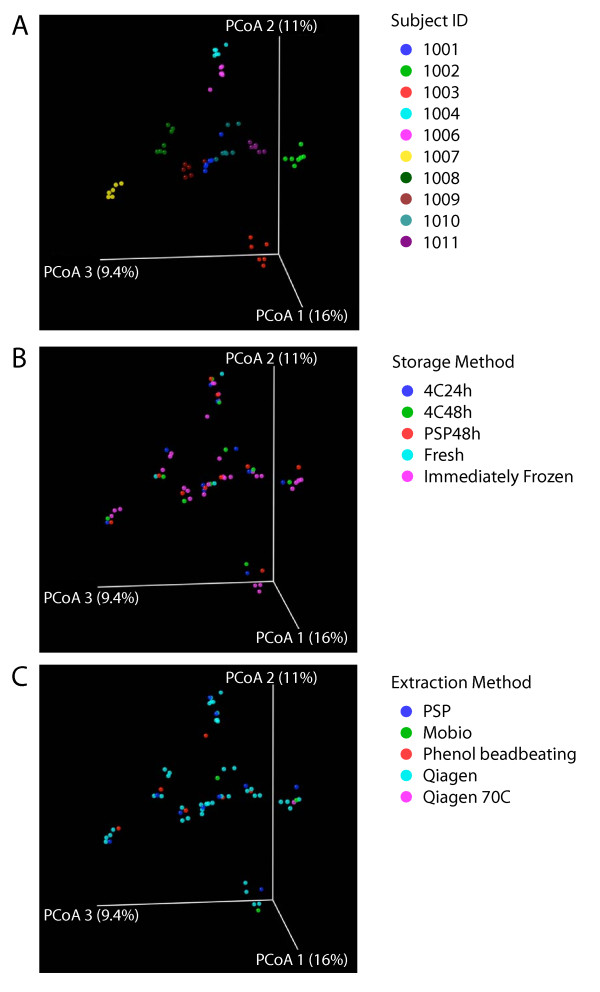
**Comparison of the presence or absence of different bacterial taxa under the different storage conditions or DNA isolation methods tested using unweighted UniFrac**. Unweighted UniFrac was used to generate a matrix of pairwise distances between communities, then a scatterplot was generated from the matrix of distances using Principal Coordinate Analysis. The same scatterplot is shown in A)-C), but colored by subject A), storage method B), or extraction method C). The P-values cited in the text were generated using distances from the original UniFrac matrix.

**Figure 4:**
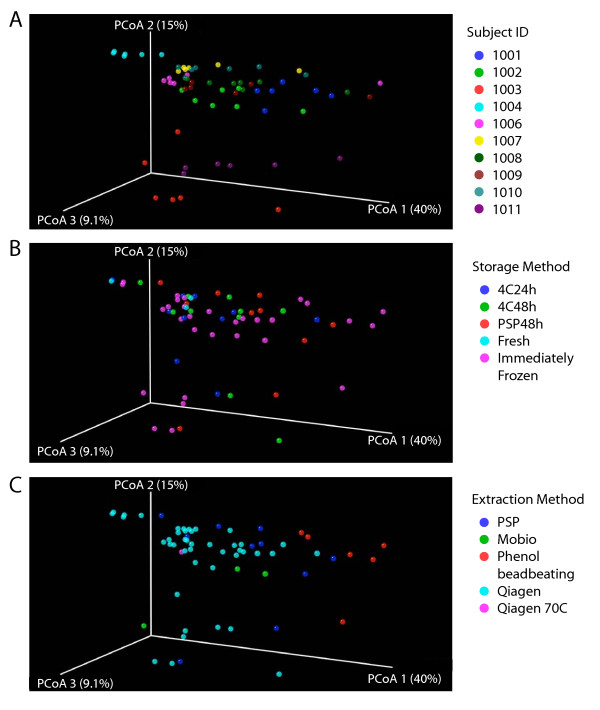
**Comparison of the relative abundance of different bacterial taxa under the conditions tested using weighted UniFrac**. Weighted UniFrac was used to generate a matrix of pairwise distances between communities, then a scatterplot was generated from the matrix of distances using Principal Coordinate Analysis. The same scatterplot is shown in A)-C), but colored by subject A), storage method B), or extraction method C). The P-values cited in the text were generated using distances from the original UniFrac matrix.

The 16S rRNA gene sequence reads from the 57 communities were aligned to generate a phylogenetic tree using FastTree2 [35]. Communities were then compared in a pair-wise fashion by means of the UniFrac distance metric, which quantifies the proportion of the branch length on the tree unique to each community in each pair. Pairwise UniFrac distances were used to generate a matrix of all distances between pairs of communities, and principal coordinate analysis used for the cluster analysis (Figures 3 and 4). All steps were carried out in an automated fashion within QIIME [36]. UniFrac analysis was carried out either unweighted, using only presence-absence information, or weighted, which takes in to account the relative proportions of each group.

The UniFrac distances matrices were first used to test for significant differences between "gold standard" samples (taken 1 cm apart on a single piece of stool). We tested the difference between pairs using distance based NP-MANOVA, which yielded p = 0.085 for unweighted UniFrac and p = 0.197 for weighted UniFrac. Thus the two gold standards were not significantly different.

Figure 3A shows the unweighted UniFrac analysis colored to distinguish communities from the 10 individuals studied. Figure 3B shows the same scatter plot colored by storage method, and Figure 3C shows the plot colored by extraction method. The data emphasizes that individuals differ substantially from each other, and that storage and extraction methods have less pronounced effects. Also present in each individual cluster are the two replicates from 1 cm apart, emphasizing the reproducibility of the method. Statistical analysis was carried out by asking whether unweighted UniFrac distances were greater within groups than between groups, then 10,000 label permutations were used to generate an empirical P-value. Clustering by subject was highly significant (P < 0.0001). No significance was seen for clustering by extraction method (P = 0.16) or storage method (P = 0.98). We conclude that overall clustering, when analyzed for presence or absence of different bacterial groups, is dominated by differences between individuals.

Figure 4 shows the weighted UniFrac analysis, which takes into account information on relative abundance, comparing the influence of individual of origin (Figure 4A), extraction method (Figure 4B), or storage method (Figure 4C). Again the differences among subjects were highly significant (P < 0.0001), but now the differences due to extraction methods were also significant (P = 0.001). Differences due to storage method were not significant. Thus when the proportional representation of different taxa is taken in to account, both the subject of origin and the extraction method exert significant effects.

We next investigated whether significant clustering could be detected when each extraction method was compared individually to the collection of other extraction methods. Again UniFrac distances were analyzed for within group and between group comparisons, and an empirical P-value generated from 10,000 permutations. No significant clustering was seen in the unweighted analysis. However, using weighted UniFrac significant clustering was seen for the phenol-bead beating method (P = 0.041) and the Qiagen method (P = 0.0014). The strong effect of the Qiagen method was driven in part by the fact that the most samples were analyzed using the Qiagen method, so the sample size was relatively large. Comparison of each method to the two gold standards using NP-MANOVA showed that the phenol bead beating and PSP methods both achieved p = 0.001. Given the significant differences for these methods, we investigated which taxa were most strongly affected, and found that in both cases the recovery of *Firmicutes *was increased while the recovery of *Bacteriodetes *was decreased (asterisks in Figure 2A and 2D), possibly a result of improved lysis of *Firmicutes *with these methods (discussed below).

### Comparison of the 454 GS FLX versus 454 Titanium sequencing methods and the effect of 16S rRNA gene region sequenced

**Figure 5:**
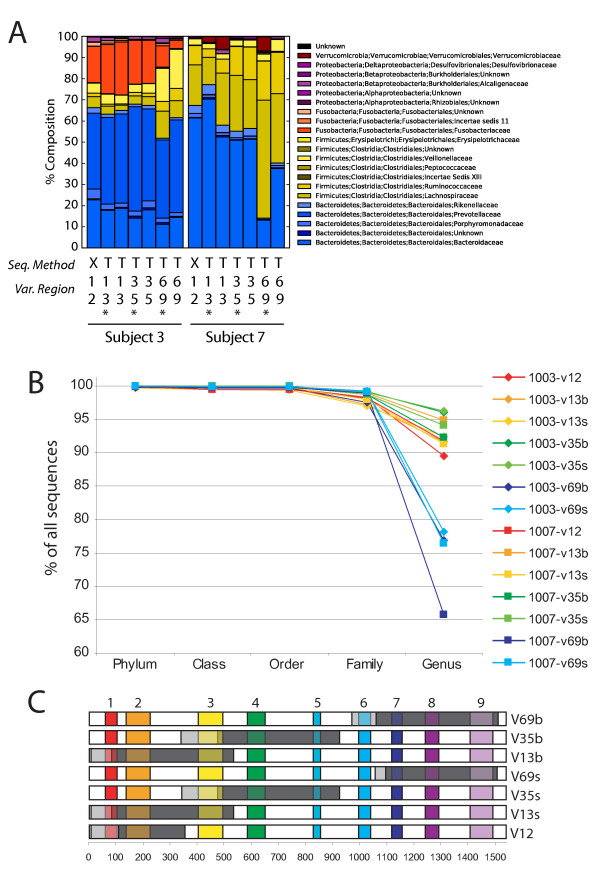
**Analysis of community composition determined using different recovery and sequencing strategies**. A) Results of analysis of Subjects 3 and 7 are shown comparing sequencing using 454/Roche GS FLX versus Titanium, and use of different variable region primers. To characterize the Titanium sequencing method, 295,946 454 Titanium sequence reads were used (Additional File 2). The 454 GS FXL reads are from the samples in Additional File 1. The percentages of different bacterial families are compared in bar graphs. "Seq. Method" indicates GS FLX ("X") or Titanium ("T"). The families present are indicated in the key beside the graphs. "Var. Region" indicates the 16S rRNA gene region amplified by each primer set (sequences used are in Additional File 4). The * indicates slightly different versions of the primers used as specified in Additional File 4. B) Percentages of sequences assigned for each primer set as a function of taxonomic level. C) Summary of regions amplified and regions sequenced for each primer set. Gray indicates the regions amplified, dark gray indicates the regions sequenced, light gray indicates regions amplified but not sequenced.

454/Roche recently introduced Titanium chemistry, which results in longer sequence reads than the GS FLX method (~450 nt versus ~260 nt). We thus wished to compare the results of taxonomic assignments for the same samples using the two methods. Two of the DNA specimens analyzed above were resequenced using the Titanium chemistry and results compared by compiling the proportions of all taxa (Figure 5A-C).

Analysis of longer 16S rRNA gene region also necessitated use of different primer pairs to amplify longer segments of the 16S rRNA gene. Several regions of the bacterial 16S rRNA gene are highly conserved, and multiple different primer sets have been used in published studies [4,16-18,37]. Previous literature has shown that 16S PCR amplification can be biased [24], so we sought to analyze this point in the context of 454/Roche pyrosequencing. To analyze the importance of primer choice for 454 Titanium pyrosequencing, we compared six primer sets, which amplified the 16S gene variable regions V1-3, V3-5, and V6-9. For each primer pair, two slightly different sequences were used. All reads were from right to left as drawn in Figure 5C, with dark gray indicating the region of sequence determination. A total of 295,946 sequence reads were used to characterize the different primers (Additional File 2). The GS FLX primers used for comparison amplified the V1-V2 region. Primer sequences are compiled in Additional File 3.

In general, communities subjected to Titanium sequencing after amplification with the V1-V3 and V3-V5 regions resembled communities analyze with GS FLX sequencing after amplification with the V1-V2 region (Figure 5A). The communities recovered with Titanium sequencing after V6-V9 primer amplification were the most discordant. The V6-V9 primers consistently showed the lowest percentage of taxonomic assignments at the genus level (Figure 5B). We note that our choice of V6-V9 primer and sequencing direction did not cover the V6 regions efficiently, so results from others focusing on the V6 region specifically may differ from those reported here. Our data indicates that when pooling data from many experiments for meta-analysis, complications may arise when mixing V6-V9 data with data from other 16S rRNA gene regions. In this study, we also compared the effects of 20 versus 30 PCR cycles and the effects of PCR product purification using gels or binding to and elution from beads. Both were found to have little effect on the results (Additional File 2 and analysis not shown).

### Comparison of recovery of 10 cloned 16S rRNA gene sequences after 454/Roche pyrosequencing

One question in analyzing microbial communities by 16S rRNA gene pyrosequencing centers on whether the amplification and sequencing methods result in recovery of sequences in proportion to their representation in the original community [24]. As a first step in addressing this issue, we prepared and analyzed a mock DNA community composed of ten bacterial plasmids encoding near full length 16S rRNA gene fragments. The mixture was PCR amplified using primers that amplified the V1-V2 region of the 16S rRNA gene, and sequences were acquired using the GS FLX technology. Sequences were acquired for both an even mixture of the ten plasmids, and a staggered mixture (a total of 28,161 sequence reads; Additional File 4).

**Figure 6:**
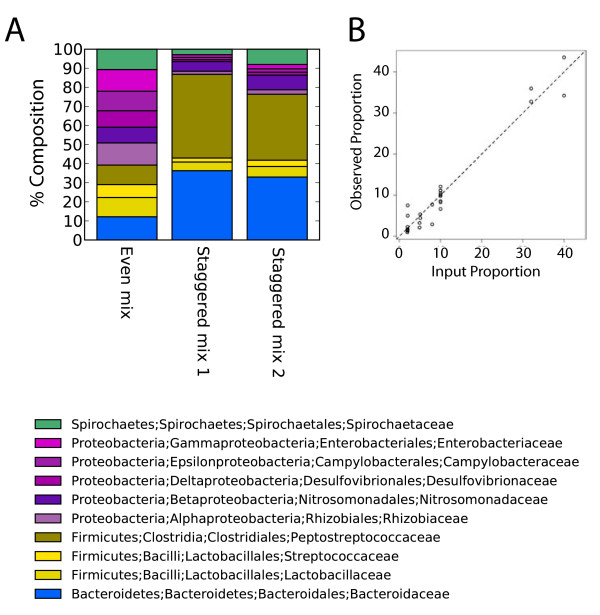
**Analysis of recovery efficiency after 454/Roche GS FLX sequencing of a cloned DNA mock community**. A) Bar graph illustrating proportional recovery of 16S rRNA gene pyrosequence reads from a plasmid DNA mock community. A total of 28,161 sequence reads were used for this analysis (Additional File 4). Each of the 10 templates consisted of a bacterial 16S rRNA gene sequence cloned in a bacterial plasmid. "Even mix" indicates that the same copy number for each of the 10 templates was used in the amplification reaction. "Staggered mix" indicates different amounts. The "Staggered mix 2" sample was amplified with a different polymerase mixture (Promega's GreenTaq Master Mix, Madison, WI) instead of AmpliTaq which was used in all other experiments, revealing that the two mixtures yielded similar results. The taxonomic assignments in this and subsequent figures are color coded as indicated. B) Scatter plot comparing the theoretical proportion of each input sequences (x-axis) to the proportions inferred from 454 GS FLX sequence data (y-axis).

Figure 6A shows the distribution of sequences. In the even mixture, all ten sequences were recovered in roughly equal proportions, and the staggered communities showed differential recoveries in the expected directions. The two staggered mock communities were sequenced after amplification with two different DNA polymerase mixtures (GreenTaq and AmpliTaq), which did not result in major differences. Figure 6B compares the input and observed proportions for both the even and staggered communities, showing that recovery was close to the input proportions (P < 0.0001). Thus we conclude that the pyrosequencing procedure used here resulted in proportions of sequence reads that closely matched the known input when cloned 16S rRNA genes are used as PCR templates.

## Discussion

Many studies have linked the composition and dynamics of the human microbiome with health and disease. Because of the immense differences in the gut microbiome among individuals, large sample sizes are often needed to correlate microbiome composition with biological variables such as disease states [4,5,7,27,38]. We have thus conducted a detailed investigation of methods for sampling and analyzing fecal microbiome samples, with the goal of identifying optimal methods for analyzing large numbers of samples. We studied the following issues: 1) methods for storing feces prior to analysis, which is critical to the feasibility of sample collection on a large scale; 2) the effects of DNA purification from feces by different methods; 3) the effects of sequence analysis using shorter versus longer pyrosequence reads (454/Roche GS FLX standard versus Titanium chemistry); 4) the influence of amplicons querying different variable regions of the 16S rRNA gene; and 5) the efficiency of recovery of different 16S rRNA gene sequences from a cloned 16S rRNA gene mock community. Our findings allow us to make several recommendations for analysis of the gut microbiome.

We stored replicate samples on ice for various times prior to freezing or at room temperature in PSP, then compared their composition to replicates that were immediately frozen (our "gold standard"). Storage on ice for up to 48 hours prior to freezing did not result in detectable differences in bacterial communities as compared to immediately frozen gold standard samples. Slight differences were seen between replicated gold standard samples, which could be due either to variations introduced during sample workup and analysis or geographic variations in the composition of the stool specimen itself. The PSP method has several advantages, including storage of fecal specimens at room temperature for up to 48 hours, the use of a self-contained storage and isolation tubes, and a greater DNA yield than other isolation methods. No method of storage correlated with communities that showed a statistically significant difference in composition from the collection of communities from each subject. We thus propose that the fecal storage method used may be chosen based on convenience of sample collection.

In contrast, the method used for DNA isolation did have a significant effect. The phenol-bead beating and PSP methods led to recovery of a greater proportion of *Firmicutes*, as shown by the weighted UniFrac analysis. The unweighted analysis, based on presence-absence information only, did not show a significant difference, indicating that the alterations were in proportions of bacterial taxa detected, and not their presence or absence (at least at the sampling depth used here). This emphasizes that where possible it is attractive to use unweighted analysis of bacterial communities, since this is less sensitive to details of the methods used for DNA isolation. We speculate that the phenol-bead beating and PSP methods led to improved lysis of bacteria with tough cell walls (the name "*Firmicute*" is derived from *firmus *for strong and *cutis *for skin).

In additional analyses, we showed that use of the 454 GS FLX versus the Titanium sequencing method did not strongly affect the conclusions. Previous literature has established that amplification of 16S rDNA gene fragments can be biased [24], so we sought to analyze this point in the context of 454/Roche pyrosequencing because there has been some controversy on optimal regions [8,14,23,25,37]. We did find that the choice of 16S rRNA gene region used for analysis had a noticeable effect, with the V6-V9 region representing an outlier. In the primer study our sample size was smaller than for studies of stool storage and DNA isolation, so we can only comment on possible trends in the primer test data. The V6-V9 set yielded the lowest proportion of calls at the genus level, though proportions were similar to other sets at higher taxonomic levels. Our selection of primers and sequencing direction resulted in incomplete coverage of the V6 region, possibly explaining poor performance by this amplicon (though see also [23,39]).

The results with the cloned DNA mock community were encouraging, showing roughly proportional recovery of the mixed 16S rRNA gene plasmid sequences over a wide range of relative abundance, though we note that the range of abundance of bacteria in stool may be even greater. This supports the idea that the sequencing method used is suitable for quantifying the composition of complex bacterial communities, but some caution is warranted. It will be useful to compare mock DNA communities made from genomic DNA specimens rather than plasmids containing cloned 16S rRNA gene sequences, and also mock communities of whole organisms. It may well be more difficult to obtain proportional representation in more demanding tests.

## Conclusions

Based on the data presented in this report we can make the following recommendations for studying the gut microbiome from human fecal samples via deep sequencing. i) The fecal storage method can be chosen based on experimental convenience, because different storage methods had little effect on the variations in community composition compared to the variation between individuals. ii) The DNA isolation method used did have a strong effect, with the phenol-bead beating and PSP methods constituting outliers. Thus we suggest that where possible communities should be compared where either all were purified using phenol-bead beating or PSP, or all were purified using one of the other methods. In cases where it is important to know the exact proportions of *Firmicutes*, it may be best to use the phenol-bead beating or PSP methods. iii) Use of either 454 GS FLX or 454 Titanium yielded similar patterns dominated by the subject of origin, so either sequencing method can be used depending again on convenience. iv) When carrying out comparisons among multiple data sets it is important to be aware of differences among primer regions, and if possible to avoid mixing data from the v6-v9 region with data from other regions. v) The differences among subjects was the most prominent source of variation among communities. Consequently, any attempt to detect the effects of additional factors on microbiome composition, such as disease state, diet, drug use, etc., will need to take in to account the substantial variation among individuals.

## Methods

### Sample collection

Ten healthy adult volunteers (at least 18 years old) were recruited to provide a single stool sample within the Center for Clinical and Translational Research at the Hospital of the University of Pennsylvania. Exclusion criteria included having had diarrhea within one week prior to the sample collection, consumption of any antibiotics within four weeks prior to sample collection, or any prior diagnosis with inflammatory bowel disease, irritable bowel syndrome, celiac sprue, or other chronic inflammatory diseases of the intestines.

After providing informed consent, each participant completed a brief survey describing their medical history and demographic characteristics. Each participant provided a single stool specimen. All specimens were collected using a collection hat that separated the fecal content from urine or the toilet water.

From the specimen provided, a research coordinator immediately removed six samples from the surface of the specimen. Samples 2 through 6 were obtained to be at least 1 cm away from the location of the first sample. All samples were collected in a Faeces Container with Screw Cap (Cat#80.734.001, Sarstedt, Newton, NC) and the sample was leveled with a wooden spatula. The first three samples were placed in empty vials and immediately stored at -80°C. Two specimens were placed in empty tubes and stored in a Styrofoam cooler filled with ice packs. These specimens were transferred to a -80°C freezer after 24 hours and 48 hours, respectively. The final sample was placed in a vial filled with stool stabilizer from the PSP SPIN Stool DNA Plus kit (Invitek). The specimen was shaken but the specimen was not fully dissolved into the stabilizer solution. After 48 hours of storage at room temperature, the specimen was transferred to a -80°C freezer. Three patients had an extra sample collected and processed immediately. Storage times at -80°C ranged from 0-137 days; time of storage at -80°C had no discernable effect on the sequencing results.

The consistency of the stool sample was characterized using the Bristol Stool Scale [40].

### DNA isolation, PCR amplification, and amplicon purification

DNA was isolated from approximately 200 mg of stool using three different commercially-available kits: QIAamp DNA Stool Minikit (Cat#51504, Qiagen, Valencia, CA), PSP Spin Stool DNA Plus Kit (Cat#10381102, Invitek, Berlin, Germany), MoBio PowerSoil DNA Isolation Kit (Cat#12888-05, Mo Bio Laboratories, Carlsbad, CA), all of which are widely used in microbiome studies. DNA was isolated exactly as per the manufactures' instructions for both the QIAamp and PSP kits except for a 95°C lysis incubation for 5 minutes, instead of the 70°C recommended for the QIAamp kit. For isolation using the Mo Bio kit, the stool sample was vortexed to homogeneity in 1 ml of Mo Bio Lysis Buffer, centrifuged at 1500 rcf for 5 minutes at room temperature. The supernatant was then transferred to the Mo Bio PowerBead tube, incubated for 10 minutes at 65°C, then 95°C for an additional 10 minutes, followed by gentle vortexing to disperse the sample in the PowerBead solution. DNA was then isolated as per the manufacturer's instructions.

For the phenol/bead beating method, the protocol consisted of a re-suspension/disruption and lysis step that was performed prior to purification using the QIAamp Stool Kit. The frozen stool sample was placed within a MoBio 0.7 mm garnet bead tube (Cat# 13123-50 Mo Bio Laboratories, Carlsbad, CA), to which 0.5 ml of Tris equilibrated (pH 8.0) Phenol: Chloroform: IsoAmyl alcohol (25:24:1) (Cat# P3803, Sigma-Aldrich, St. Louis, MO) was added, and the remaining volume was filled up with buffer ASL from the QIAamp Stool Kit (approximately 0.9 ml). The sample was mechanically disrupted by bead beating using a MiniBeadBeater-16 (Cat# 607, Biospec, Bartlesville, OK) for 1 minute. The resulting homogenate was incubated at 95°C for 5 minutes and centrifuged at 13000G for 1 minute to separate the aqueous and phenolic phases. The aqueous phase was transferred to a new 2 ml microcentrifure tube and the volume was completed to 1.2 ml with buffer ASL. One QIAamp Stool Kit inhibitX tablet was added to this lysate and homogenized according to manufacturer specifications. The remaining of the procedure was followed according to the QIAamp Stool Kit pathogen detection protocol.

After quantification by spectrophotometry, 100 ng of DNA was amplified with barcoded primers using 2.5 units of AmpliTaq (Cat# N8080161, ABI, Foster City, CA) in a reaction buffer containing 25 mM MgCl_2_, 1% Triton, 10 mM dNTPs, and 10 mg/ml BSA (Cat #B90015, New England Biolabs, Ipswich, MA) [18]. PCR was performed on an ABI 2720 Thermocycler using the following conditions: Initial denaturing at 95°C for 5 minutes followed by 20 cycles of 95°C × 30 seconds, 56°C × 30 seconds, and 72°C × 1 minute 30 seconds. The reaction was terminated after an 8 minute extension at 72°C. The amplicons from each DNA sample, which was amplified in quadruplicate, were pooled and gel purified using an 0.8% agarose gel and a QIAquick Gel Extraction Kit (Cat# 28704, Qiagen) per the manufacturer's instructions.

### Defined DNA community composition

Two defined DNA mixture were created using 10 different plasmids, each containing a near full length 16S rDNA amplicon, obtained using primers BSF8 and BSR1541. One mixture had an equal amount of each plasmid and one was staggered to contain different proportions of each clone. The strains and proportions on the Staggered mix are: *Clostridium dificile *(ATCC#: BAA-1382) - 39.99%, *Bacteroides fragilis *(ATCC#: 25285) - 32.01%, *Streptococcus pneumoniae *(ATCC#: BAA_334) - 4.92%, *Desulfovibrio vulgaris *(ATCC#: 29579) - 1.95%, *Campylobacter jejunii *(ATCC#: 700819) - 2.03%, *Rhizobium vitis *(ATCC#: BAA_846) - 2.00%, *Lactobacillus delbruekii *(ATCC#: BAA-365) - 5.06%, *Escherichia coli *HB101 - 2.01%, *Treponema sp*. (macaque stool clone) - 7.97%, and *Nitrosomonas sp*. (environmental clone) - 2.04%. Clones were made using the Topo-XL kit (Cat# K4700-20, Invitrogen, Carlsbad, CA). Two polymerases were tested for the Staggered mix, AmpliTaq (as used for stool DNA samples) and GreenTaq (Promega, Madison, WI) as per manufacturer instructions. The PCR cycling conditions were the same as described for the stool sample DNA.

### 454/Roche sequencing methods

Purified amplicon DNAs were quantified using Quant-iT PicoGreen kit (cat# P7589, Invitrogen, Carlsbad, CA) and pooled for pyrosequencing. Pyrosequencing using the 454/Roche GS FLX chemistry was carried out according to the manufacturer's instructions. Pyrosequencing using the Titanium method was carried out using the Titanium genomic kit. Primers for PCR amplification of rDNA gene segments are in Additional File 3. The rDNA region amplified with V1-V2 primers used for FLX sequencing is contained within the regions amplified with the V1-V3 primers used for Titanium sequencing.

Pyrosequence reads were uploaded into QIIME and processed as described (Caporaso et al., 2010). Briefly, QIIME accepts as input bar coded 16S rRNA gene sequences, classifies them using the RDP classifier [23], aligns them using Pynast [31], constructs phylogenetic trees using FastTree2, calculates UniFrac distances, and generates data summaries of the proportions of taxa present and PCoA plots based on UniFrac distances. We used 97% OTUs in the analysis. For the RDP classifier, we required >50% confidence for all calls.

Accession numbers for sequences determined here are in Additional File 5.

### Statistical methods

Clinical characteristics were compared as median, range, counts and percentages. For analysis in Figures 1 and 2, no corrections for multiple comparisons were applied. UniFrac [33,34,41] was used to generate distances between all pairs of communities; both weighted and unweighted UniFrac were used in the analyses. Statistical analysis was carried out by comparing distances within groups to distances between groups. Comparisons were summarized using the t-statistic and significance assessed using 10,000 label permutations. Clustering was visualized for weighted and unweighted UniFrac data using principal coordinates analysis.

We use the distance based Permutational Multivariate Analysis of Variance (NPMANOVA) to perform overall test of the difference between the two gold standards (samples taken 1 cm apart from the same piece of stool) and between gold standards and other sampling methods using both the weighted and unweighted UniFrac distance matrix. If the overall test gave significant results, then we used signed rank test on the proportion data to pinpoint the taxonomic groups that showed significant differences in abundance between the two sampling methods.

## Authors' contributions

GDW, JDL, CH, RK, KB, HL, and FDB conceived, directed, and carried out the study; YYC and JH prepared samples for sequence analysis; RB and LN acquired samples, and JC, HL, GDW, JL, CH, KB, RK and FDB. analyzed the data. All authors have read and approved the final manuscript.

## Supplementary Material

Additional file 1 **Table S1. Samples analyzed in the study of methods for storage and DNA isolation**. This table summarizes the samples studied comparing methods for storage and DNA isolation. Click here for file

Additional file 2 **Table S2. Samples analyzed in the study of variable region primers**. This table summarizes the samples used specifically in the analysis of different variable region primers. Click here for file

Additional file 3 **Table S3. Sequences of primers used for amplification**. This table contains the sequences of primers used for PCR amplification. Click here for file

Additional file 4 **Table S4. Samples analyzed in the study of the cloned DNA mock community**. This table summarizes the samples used in the study of the cloned DNA mock community. Click here for file
